# Conceptualisation and application of implementation strength in public health programmes within health systems: a systematic review

**DOI:** 10.1186/s12913-026-14856-w

**Published:** 2026-06-09

**Authors:** Waqas Hameed, Bilal Iqbal Avan, Mudassir Uddin

**Affiliations:** 1 Department of Statistics, University of Karachi, Karachi, Sindh Pakistan; 2 Department of Community Health Sciences, Aga Khan University, Karachi, Pakistan; 3 Department of Population Health, London School of Hygiene and Tropical Medicine, London, UK

**Keywords:** Health systems, Implementation research, Implementation strength, Implementation intensity, Scale up, Health programme, Healthcare services, Monitoring

## Abstract

**Background:**

Implementation strength (IS), a cardinal concept in the field of implementation science, is used to assess how the implementation of health services and specific health programme influences health outcomes. It offers a unique opportunity to determine how much implementation effort is required to achieve a meaningful level of change in outcomes. Despite its growing use, a lack of conceptual clarity has led to inconsistent measurement and sub-optimal application. We explored how IS is defined, measured, and associated with outcomes, and proposed a set of recommendations for its future use.

**Methods:**

Using an integrated approach combining systematic review and principles of concept analysis, a comprehensive search of PubMed, Scopus, ERIC, and Google Scholar was conducted up to July, 2025. Peer-reviewed theoretical and empirical studies conducted within health systems—regardless of study design, location, or population were eligible for inclusion. JBI tools were used for quality assessment. Data were synthesised using a content analysis approach.

**Findings:**

IS is often vaguely defined, with mismatches between its definitions and how it is measured. The concept has primarily been applied to health programmes implemented in real-life settings; however, no study has applied it to routine healthcare services. Studies conceptually-grounded in 2011-13 IS guidance differed notably from their counterparts: they used fewer but more relevant indicators, advanced statistical methods for composite indices, and examined dose-response relationship. Yet, most studies overlooked broader programme components outlined in the WHO monitoring & evaluation framework. Measurement practices varied widely and often conflating IS with implementation fidelity. Few studies analysed the relationship between IS and outcomes, but those that did reported a strong positive association. While half of the studies were of high quality, most lacked a reliable and valid composite IS index.

**Conclusion:**

Our review comprehensively mapped existing confusion surrounding the conceptualisation of IS, identified shortcomings in its application, and offers a scientifically grounded resolution aimed at standardising its conceptualization and application, thereby enabling consistent comparisons across settings. Strong evidence of a positive relationship between IS and health system outcomes reinforces the value and potential of this approach in strengthening health systems. Future research should focus on developing standardised and validated IS indices across health themes, along with effective and feasible approaches for integrating them into routine health systems for use by health administrators.

**Clinical trial number:**

Not applicable.

**Supplementary Information:**

The online version contains supplementary material available at 10.1186/s12913-026-14856-w.

## Strengths and limitation of this study

- To the best of our knowledge, this is the first comprehensive systematic review globally to provide clarification on the concept of implementation strength within a health system context.
- Our review identified and critically discussed key gaps in the conceptualization and application of IS, and also revealed a strong positive relationship between IS and health outcomes.
- It offers guidance for scholars and practitioners by providing a comprehensive definition of the concept along with a scientifically grounded guidelines to standardise its conceptualisation and application across contexts.
- Our review included only English-language articles due to our familiarity with the language, which may introduce potential publication bias and limit generalisability and contextual insights.

**Supplementary Information:**

The online version contains supplementary material available at 10.1186/s12913-026-14856-w.

## Background

Strengthening health systems often involves scaling up evidence-based, high-impact public health interventions to improve population health [1, 2]. However, expanding the benefits of successful pilot projects to serve a larger population equitably and sustainably is a major challenge in global health [3]. Interventions with proven effectiveness often yield inconsistent outcomes when implemented in real-world settings [4–6], primarily due to inadequate implementation—commonly referred to as an ‘empty scale-up’ [7]. This is because innovations in health services are typically pilot-tested in controlled environments with specialised organisational, financial and human resources, which are often not available when innovations are integrated into health systems at scale. Therefore, scaling-up requires deliberate planning, effective management, and appropriate resource allocation [2], with sufficient flexibility and adaptability to evolving conditions throughout implementation in the long term.

Implementation science is a distinct field that focuses on the systematic integration of evidence-based interventions into real-world practice [8, 9] by uncovering the contextual and systemic factors that influence the success or failure of programmes and policies [8, 9]. Within the paradigm of implementation science, implementation strength (IS) has emerged as a cardinal concept in the global health lexicon. It offers a unique perspective on the large-scale implementation of evidence-based interventions [10–12] by quantifying implementation efforts and examining how these efforts influence health system outcomes [10, 13, 14]. This, in turn, helps guide implementers in determining the level of effort required to achieve meaningful changes in service coverage or health outcomes.

Over the past decade, the concept of IS has gained significant traction and has been increasingly applied as a performance monitoring technique in global health systems research. Some researchers have used it descriptively to assess implementation efforts across different domains [15]; while others have examined factors associated with implementation efforts [13, 16] and the relationship between implementation efforts and health outcomes [17, 18]. Reportedly, the measurement of IS typically follows the input-process-output (IPO) framework [11], involving key components of the programme such as leadership and human resources, and information systems, as well as the processes underpinning service delivery, such as their availability and readiness [11]. Importantly, while the concept is, in principle, equally applicable for monitoring routine health services within health systems, it has been predominantly used with a programme-focused approach globally.

Despite growing recognition and utilisation of IS, its operationalisation and measurement remain ambiguous [10, 19]. The lack of conceptual clarity—particularly in distinguishing it from implementation fidelity—along with limited guidance on standardised assessment methodologies [11], has caused inconsistencies in its application and resulting confusion (Table 1). Two primary factors contribute to this issue. First, the idiosyncratic use of IS-related terms involves homonymy (the same term having multiple meanings), synonymy (different terms conveying the same meaning), and instability (the meaning and use of terms evolve over time unpredictably) [20]. Second, published descriptions of the concept often lack sufficient detail to enable either scientific or real-world replication [21]. Together, these deficiencies complicate the acquisition and interpretation of knowledge, hinder research syntheses such as systematic reviews and meta-analyses, limit replication in both research and practice, and ultimately obstruct the translation and application of empirical studies that could otherwise inform and improve implementation processes [21–23].

**Table 1:** Conceptual differences between implementation strength and implementation fidelity

S.no	Implementation strength [10, 11, 13, 19]	Implementation fidelity [24, 25]
1	Quantifies programme implementation efforts	Evaluates adherence to the established protocol or plan
2	Reflects ‘strength/intensity’ of implementation	Reflects ‘integrity’ or ‘faithfulness’ of implementation
3	Theoretically, the measure has an infinite range on the higher end – i.e. from $\:0\:to\:\infty\:$. The higher score indicates higher strength	Theoretically, the measure has a finite range – i.e. from 0 to 1 (on probability scale), where higher probability indicates higher adherence, with 1 indicating perfect adherence
4	Primarily used for monitoring of programme implementation in real-life settings	Primarily used for monitoring the implementation (of interventions) in research settings
5	Principally takes into account all input, process and output level activities or support	Primarily assesses procedural adherence to intervention in terms of content, coverage/dose, frequency and duration
6	Understand variability in implementation efforts and establish dose-response relationship with programme targets	Understand implementation deficiencies and the extent to which these may dilute the effect of research interventions on study outcomes, an indication of type-III error

While some efforts have been made to address the measurement aspects of the IS concept [10], there remains a critical necessity persists to holistically explicate the concept of IS and its application. This comprehensive elucidation involves not only distinguishing it from cognate notions such as implementation fidelity in terms of purpose and use, but also establishing a standard approach for its measurement and interpretation in the context of health system research. Addressing this gap requires a comprehensive concept analysis, a systematic approach to clarifying the meaning, purpose, and use of a concept. It identifies key components, prerequisites, and consequences while facilitating the development of an operational definition—essential for enhancing both understanding and practical application. This foundational work is crucial, as the lack of conceptual clarity can lead to methodological weaknesses, compromising the validity, reliability, and overall strength of the empirical evidence in this field [26].

To the best of our knowledge, the existing literature does not consolidate a foundational understanding of the IS concept or establish a clear basis for future research by integrating systematic review and concept analysis methods. With the overarching goal of improving the conceptual clarity of IS for its accurate characterisation, measurement, and application, we conducted a systematic review guided by the following research objectives:

## **Objective**

Explore the conceptualisation, components, and measurement of IS, and to examine whether evidence exists that interventions with higher IS lead to better outcomes.

### **Specific sub-questions:**

- How IS is conceptualised, and what components are used in health system research?
- How the concept of IS is measured, and what types of statistical techniques are commonly employed in its measurement?
- How the measure of IS is analysed, and does the existing evidence support the hypothesised positive relationship between IS and health outcomes?

## Methods

### Design

We adapted the widely recognised Walker and Avant’s approach [27] to concept analysis, incorporating the following steps: (1) characterisation of the concept and its purpose, (2) determining key components of the concept, (3) measurement approaches, and (4) use of concept and its consequences in terms of the relationship with outcomes. The findings of this review are critically discussed, followed by a proposed definition and a set of recommendations for its operationalization in future research. Despite its widespread use, the Walker and Avant method does not offer a systematic or rigorous procedure for identifying and selecting relevant literature. Hence, we adopted an integrated approach that combines the strengths of systematic review and concept analysis to address the limitations of each method, thereby enhancing the rigour and transparency of the findings [28].

This systematic review adhered to the international Preferred Reporting Items for Systematic Reviews and Meta-Analyses (PRISMA) guidelines [27, 29]. In addition, the methods followed the relevant guidelines from the Cochrane Handbook where applicable. To ensure transparency, an a priori protocol for this study was registered with PROSPERO (Registration ID: CRD42025629408). The protocol can be accessed from PROSPERO.

### Search strategy

With the assistance of health professionals who specialize in health systems research and systematic reviews, we refined the search strategy originally developed for a literature review conducted in 2012 [11]. The search was conducted across four databases: PubMed, Scopus, Eric, and GoogleScholar until July 12, 2025. A detailed search strategy was developed comprising three major components of words phrases: (1) implementation, (2) measure* or evaluation* or assess* or examine*, and (3) strength or intensity or strength or degree or level or extent or rate. We also searched for grey literature using google search and pertinent websites, including WHO’s official website, website of different UN agencies, Popline, and others. Finally, to minimize selection bias, we conducted a thorough manual search by examining the reference lists of shortlisted articles. This approach ensured that all potential sources were considered, reducing the likelihood of oversight or exclusion of relevant studies. The complete search strategy per database is provided in supplementary file 1.

### Eligibility criteria

Our study selection followed the Population, Context, and Concept (PCC) criteria [30]. The population included all beneficiaries of research interventions or programmes applying the IS concept, regardless of demographic characteristics. The context encompassed any level of the health system (primary, secondary, or tertiary) in any geographic location. The concept focused on theoretical and empirical studies contributing to the conceptualisation, measurement, or application of IS.

We imposed no restrictions on study design, participant type, or type of intervention (e.g., mental health, maternal and child health, behavioural sciences, nursing). Studies using both primary and secondary data were included. In summary, eligible studies addressed at least one of the following within health systems: (a) description, significance, or use of the IS concept; (b) IS measurement methodologies; or (c) links between IS and outcomes. All studies involving human subjects, published in English up to July 12, 2025, were included, with no start date limit. In view of the mixed evidence regarding the effect of excluding non-English-language studies on review findings and conclusions and due to our familiarity with the language, we confined our search to English-language studies.

### Selection of studies

All searched records were imported into Zotero software to remove duplicates. They underwent a two-stage screening process for eligibility: (a) title and abstracts and (b) full-text screening of studies that passed initial screening stage. The primary author (WH) conducted the review of all titles, abstracts, and full texts, while a random sample of 5% was cross-verified by co-authors to ensure quality assurance. Any disagreements regarding the inclusion or exclusion of studies were resolved through discussion, discourse, and collective consensus among all authors. In cases where the same study appeared in both a grey literature report and as a peer-reviewed publication, the peer-reviewed version was retained to maximise methodological rigour.

### Data extraction and synthesis

Data were extracted by the primary author (WH) into a structured excel sheet from selected full-text studies. Apart from publication details, the main characteristics included: conceptualisation (definition, purpose and guiding framework), measurement (indicators content, scale, statistical techniques for composite indices, unit of assessment, analytical approach, and psychometric properties), and consequences of the concept, including empirical evidence on the association between IS and outcome (Table 2). The accuracy of data extraction was verified by co-authors, who independently extracted and compared 5% of the selected studies.

**Table 2:** Glossary of terms

Term	Definition	Examples
Input	The resources that are used to carry out activities [31]	Financial, human, material, and infrastructure
Process	The activities or actions carried out to deliver services, measuring implementation function of health system or programme [31]	Trainings, health facilities receiving supportive supervision visits
Output	Immediate results produced by the activities and processes [31]	Number of patient treated
Components	The component parts of something are the parts that make it up. In the context of a health system, a component refers to an essential part or element that contributes to the system’s overall function [32].	Supply-side material and activities (e.g. health workers, supplies)
Pre-requisites	Pre-requisites are those events or incidents that must occur or be in place prior to the occurrence of the concept [27]	Intervention or programme, implementation variability
Consequences	Consequences are those events or incidents that occur as a result of the occurrence of the concept, the outcomes of the concept [27]	Improved health outcomes (e.g. prevalence of contraceptive, reduction in illness)

We used a deductive content analysis approach to synthesise the data. Guided by our research questions [33], the analysis was performed in three stages. To understand how IS is conceptualised, we first identified and grouped key terms from reported IS definitions into broader dimensions emerging from the data. Second, we examined multiple aspects of IS measurement. Given the health system focus of our work, we assessed the measurement components of IS using the WHO Monitoring & Evaluation Framework for health system [34]. The framework explicitly takes into account the WHO’s six building blocks of the health system [31] and Input-Process-Output aspects of the programme theory [35]. Furthermore, we reviewed data sources (e.g., surveys, HMIS), indicator types and scale, statistical methods used for calculating and categorising composite IS indices. And third, to understand how IS indices are used, we examined the units of analysis, statistical methods applied, and how IS is associated with health outcomes.

We conducted both an overall and comparative analysis, grouping studies into two categories. A study was classified as “IS-adherent” if it met at least one of two-pre-specified criteria: (a) the study explicitly cited a foundational IS guidance document - either the 2011 JHU working paper [11] or the 2012 LSHTM literature review [19] - as a conceptual reference for IS; or (b) the study was a follow-up work of one or more authors from the foundational documents. Studies meeting neither criterion were classified as “non-IS-adherent”. For each group, we compared measurement methods, commonly used statistical techniques, and the relationship between IS and outcomes. An independent t-test (Stata v16.0) was used to compare the average number of indicators used across both groups. All other data were analysed manually using Excel.

### Research quality assessment (risk of bias)

To the best of the authors’ knowledge, no appraisal tools currently exist for assessing the quality of research studies — including commentaries and perspective pieces — that describe the *conceptualisation* of a phenomenon. Given that a dose-response relationship is one of the underlying characteristics of the IS concept, risk of bias assessment was conducted only for the subset of studies that empirically examined the relationship between IS and outcomes. A total of 22 studies were assessed using Joanna Briggs Institute (JBI) tools which are widely recognized [36] and recommended by experts [37, 38]. We adapted the rating scale to capture finer details regarding quality, using the following categories: 0 = no/unclear/not reported, 1 = yes, partially, 2 = yes, completely/not applicable. Based on these assigned numeric codes, a composite score was calculated by adding-up the score for each item, where higher score indicated higher quality (or lower risk of bias) of the study. The score was then transformed to a scale of 0-100 and then divided into three categories using tercile: ‘Unacceptable’ (≤ 33.3), ‘acceptable’ (> 33.3 to ≤ 66.6), and High (> 66.6).

### Patient and public involvement

No patient involved.

## Results

**Fig. 1:**
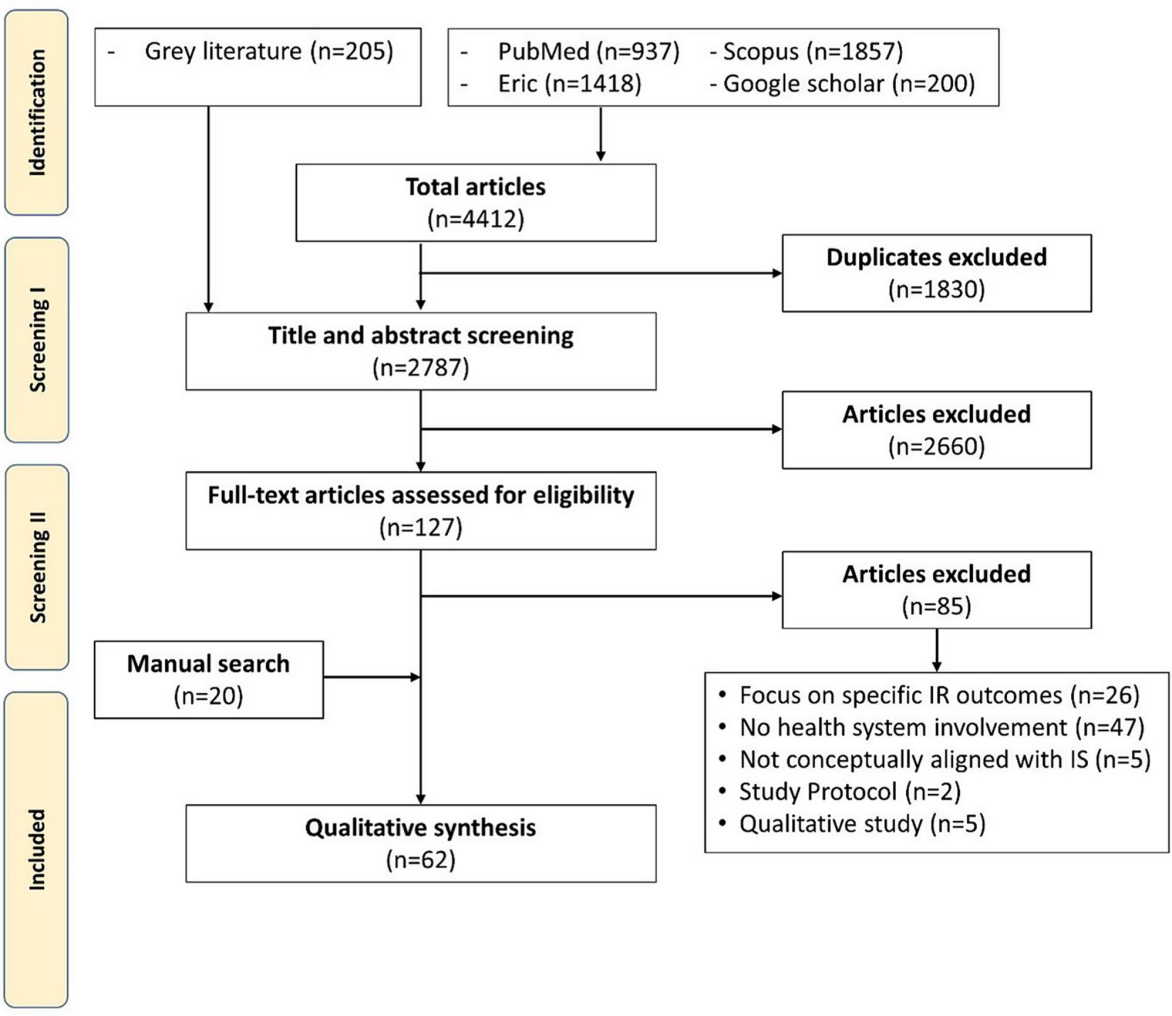
PRISMA flow chart showing results of search strategy and study selection process

The results begin with a PRISMA flowchart [39] and study characteristics, followed by key findings in three sections: conceptualisation of IS, methodological approaches to its measurement, and its use and its relationship with outcomes. The study selection process is presented in Fig. 1. A total of 4,412 articles were retrieved from the database search, which eventually came down to 62 studies after removing duplicates and eligibility screening.

### Characteristics of studies included in the review

The number of publications increased progressively over time, with the majority (72.6%, *n* = 45) of studies conducted in high- and low-income countries – nearly equal split between these income strata. About one-third (35.5%, *n* = 22) were from the Sub-Saharan African region. In terms of the thematic focus, child health was the most common area of research (36.8%, *n* = 28) followed by maternal health (17.1%, *n* = 13) (Table 3).

**Table 3:** Characteristics of included studies (*N* = 62)

Study characteristics	*n*	%
Primary Theme*		
Child health	28	36.8
Maternal health	13	17.1
Communicable, Non-Communicable and Infectious diseases	11	14.5
Health System	7	9.2
Mental Health	5	6.6
Family Planning	5	6.6
Quality of care	3	3.9
Pharmacy	3	3.9
Geriatric Health	1	1.3
**Year of publication**		
2020–2025	18	29.0
2016–2019	14	22.6
2012–2015	8	12.9
2008–2011	7	11.3
2000–2003	6	9.7
2004–2007	5	8.1
1992–1995	4	6.5
**Distribution of countries by income-strata**		
High-income	23	37.1
Low-income	22	35.5
Middle-income	11	17.7
Multi-country	3	4.8
Conceptual/Theoretical	3	4.8
**Regional distribution of countries**		
Sub-Saharan Africa	22	35.5
North America	14	22.6
Latin American and the Caribbean	8	12.9
Europe and Central Asia	6	9.7
Multi-country	5	8.1
South Asia	3	4.8
Conceptual/Theoretical	3	4.8
East Asia and Pacific	1	1.6
Middle East and North Africa	0	0.0

* Multiple themes are possible in a study

Most were conducted in the United States (24.2%, *n* = 15), followed by Ethiopia and Malawi with eight studies each (12.9%). Nearly all studies (91.9%, *n* = 57) used primary data to apply the concept of IS, while the rest were perspective papers discussing its conceptualisation and measurement. The majority (93.5%, *n* = 58) evaluated existing programmes, with only a few (6.5%, *n* = 4) focusing on research interventions. Notably, we found no study that applied the concept of IS on routine health services aiming to strengthen either public or private healthcare systems (Table 4).

**Table 4:** Context and technical characteristics of included studies

Context			Technical aspects				
Author (Year)	Country	Settings	Unit of analysis	Data Source	Primary Theme	HSP / ER^1^	Research quality^2^
**Primary research**							
Lauren A. Hinrichs-Kinney, et al. (2025)	United States	Health facilities	Health facilities	Health workers	Rehabilitation	HSP	Not assessed
Benjamin R. Brady, et al. (2024)	United States	Health facilities	Health facility	Program Managers	Health System	HSP	Not assessed
Juliana Mara Flores Bicalho et al. (2023)	Brazil	Health facilities	Health facility	Program Managers	Non-communicable disease	HSP	Not assessed
Wanessa Debôrtoli de Miranda et al. (2023)	Brazil	Health facilities	Health facility	Program Managers	Child health	HSP	Not assessed
Luay Basila et al. (2022)	Mali	State primary healthcare system	Healthcare professional	Health workers	Child Health	HSP	Not assessed
Anooj Pattnaik et al. (2022)	Malawi	State primary healthcare system	Cluster	Healthcare workers	Family Planning	HSP	High
Anooj Pattnaik et al. (2021)	Malawi	Facilities and healthcare worker	Health facility	Health facilities; Health workers	Family Planning	HSP	Not assessed
Flavio Renata Barros da Guarda et al. (2021)	Brazil	Community-based settings	Cluster	Program Managers	Health System	HSP	Not assessed
William T. Story et al. (2021)	Ethiopia	Village	Cluster	Health workers	Maternal Health; Child Health	HSP	Unacceptable
Julian Schwarz et al. (2021)	Multi-country	Hospitals	Health facility	Program Managers	Mental Health	HSP	Not assessed
Trine Damsted Rasmussen et al. (2021)	Denmark	Hospitals	Health facility	Health workers	Maternal Health; Child Health	ER	Not assessed
Xiaomeng Chen et al. (2021)	Mali	Healthcare system	Healthcare professional	Health workers	Child Health; Family Planning	HSP	Not assessed
Ikhlaq Ahmad et al. (2020)	Pakistan	Lady Health Worker Programme	Healthcare professional	Lady Health Workers	Mental Health	ER	Acceptable
Anooj Pattnaik et al. (2020)	Malawi	State primary healthcare system	Healthcare professional	Health facilities; Health workers	Family Planning	HSP	High
Ania Gorostiza et al. (2020)	Basque	Hospitals	Patient	Health facilities	Non-communicable disease	HSP	High
Brener Santos Silva et al. (2020)	Brazil	Vaccination Centres	Health facility	Health workers	Child Health	HSP	Not assessed
Samuel Chipokosa et al. (2019)	Malawi	Healthcare worker	Healthcare professional; District	Health facilities; Health workers	Family Planning	HSP	Not assessed
Kirsten Zalisk et al. (2019)	Malawi	State primary healthcare system	Healthcare professional	Health workers	Child Health	HSP	Acceptable
Bereket Mathewos et al. (2019)	Ethiopia	State primary healthcare system	Healthcare professional	Health workers	Maternal Health; Child Health	HSP	Not assessed
Sofie Østergaard Jaspers et al. (2019)	Denmark	Hospitals	Health facility	Health workers	Mental Health	ER	Not assessed
Nicola Vousden et al. (2019)	Multi-country	Secondary or tertiary Hospital	Health facility	Health workers	Maternal Health	ER	Acceptable
Voodgt-Pruis HR et al. (2019)	Netherland	Health facilities	Health facilities	Health workers	Non-communicable disease	HSP	High
Zufan Abera Damtew et al. (2018)	Ethiopia	Village	Cluster	Health workers	Maternal Health; Child Health	HSP	High
Ali Mehryar Karim et al. (2018)	Ethiopia	Facilities and healthcare worker	Cluster	Health workers	Maternal Health; Child Health	HSP	High
Gizachew Tadele Tiruneh et al. (2018)	Ethiopia	Healthcare system	Health facility	Health facilities; Health workers	Maternal Health; Child Health	HSP	Acceptable
Nathan P Miller et al. (2016)	Ethiopia	State primary healthcare system	Healthcare professional	Health facilities; Health workers	Child Health	HSP	High
Agbessi Amouzou et al. (2016)	Malawi	State primary healthcare system	District	Health workers	Child Health	HSP	Acceptable
Srofenyoh EK et al. (2016)	Ghana	Health facilities	Health facility	Health workers	Maternal health	HSP	Acceptable
Rebecca Heidkamp et al. (2015)	Malawi	State primary healthcare system	District	Health facilities; Health workers	Child Health	HSP	Not assessed
Elizabeth Hazel et al. (2015)	Malawi	State primary healthcare system	Healthcare professional	Health workers	Child Health	HSP	Not assessed
Gregory Sondo Kabadi (2015)	Tanzania	State primary healthcare system	District	Health workers	Maternal Health; Child Health	HSP	High
Nathan P. Miller et al. (2014)	Ethiopia	State primary healthcare system	Healthcare professional	Health workers	Child Health	HSP	Not assessed
Marie Boltz et al. (2013)	United States	Hospitals	Health facility	Health workers	Geriatric Health	HSP	Not assessed
Ali Mehryar Karim et al. (2013)	Ethiopia	Healthcare worker	Cluster	Women	Child Health	HSP	High
Eran Bendavid et al. (2012)	Multi-country	HIV programs	Country	Program Managers	Infectious disease	HSP	High
Catherine Dumartin et al. (2011)	France	Hospitals	Health facility	Health facilities	Pharmacy	HSP	Not assessed
T.K. Ryman et al. (2011)	North Sudan	Healthcare system	District	Health workers	Child Health	HSP	Unacceptable
Ruchi Sogarwal et al. (2011)	India	Healthcare system	State	Health facilities; Health workers	Infectious disease	HSP	Not assessed
A Amouzou et al. (2011)	Multi-country	Health facilities	Healthcare professional	Children	Child Health	HSP	Not assessed
Ligia Maria Vieira-da-Silva et al. (2010)	Brazil	Healthcare system	State	Health facilities; Health workers	Health System	HSP	Acceptable
Giunta N (2010)	United States	National Family Caregiver Support Program	State	Program Managers	Mental Health	HSP	Not assessed
Peter d’Abbs et al. (2008)	Australia	Community-based settings	Cluster	Health workers	Non-communicable disease	HSP	Not assessed
Jeffrey A. Alexander et al. (2006)	United States	Hospitals	Health facility	Health facilities	Quality of care	HSP	High
Tarayn A. Grizzard et al. (2006)	United States	Hospitals	Health facility	Health workers	Child Health	HSP	Not assessed
Marjorie L. Pearson et al. (2005)	United States	Hospitals	Health facility	Health workers	Non-communicable disease	HSP	Not assessed
R. Rejean Hebert et al. (2004)	Canada	Healthcare system	Cluster	Health workers	Health System	HSP	Not assessed
Heli Kansanaho et al. (2004)	Finland	Pharmacies	Pharmacies	Health workers	Pharmacy	HSP	Not assessed
Marly Aparecida E. Cosendey et al. (2003)	Brazil	Pharmacies	Health facility	Health workers	Pharmacy	HSP	Not assessed
W. Carl Cooley et al. (2003)	United States	Paediatrics Primarycare	Health facility	Health workers	Child health	HSP	Not assessed
DIANE PAULSELL et al. (2002)	United States	Early Head Start Programs - Community- and school settings	Program	Program Managers	Child Health	HSP	Not assessed
Amy E. Bonomi et al. (2002)	United States	Healthcare system	Health facility	Health workers	Non-communicable disease	HSP	Not assessed
Sunhee Lee et al. (2002)	Korea	Hospitals	Health facility	Health facilities	Quality of care	HSP	Not assessed
Robert G. Orwin (2000)	United States	Hospitals	Healthcare professional	Health facilities	Mental Health	HSP	Not assessed
Stephen M. Shortell et al. (1995)	United States	Hospitals	Health facility	Health workers	Quality of care	HSP	High
Goldman KD (1994)	United States	Organization	Healthcare professional	Program Managers	Maternal Health; Child Health	HSP	Not assessed
y Rachel Benson Gold et al. (1993)	United States	MCH Program	State	Program Managers	Maternal Health; Child Health	HSP	Not assessed
Karen Glanz et al. (1992)	United States	Physician Offices	Healthcare professional	Health workers	Non-communicable disease	HSP	Not assessed
**Theoretical / Conceptual papers**							
Avishek Hazra et al. (2022)	India	Healthcare system	Healthcare professional	N/A	Maternal Health; Child Health	HSP	Not assessed
Lara M. E. Vaz et al. (2020)	Global	Healthcare system	District	N/A	Health System	HSP	Not assessed
Sushama K.Sreedhara et al. (2018)	Global	Healthcare system	NR	N/A	Health System	HSP	Not assessed
James R M Hargreaves et al. (2016)	Global	Healthcare system	Individual, District, Country	N/A	Maternal Health; Child Health; Infectious disease; Communicable disease	HSP	High
Theresa Diaz et al. (2014)	Multi-country	State primary healthcare system	Healthcare professional	Health facilities	Child Health	HSP	Not assessed

^1^ HSP: Health Services/Promotion Programme; ER: Evaluation Research

^2^ Studies that did not examine the relationship between IS with outcomes were not assessed for research quality

### Conceptualisation

About half of the studies (50%, *n* = 31) explicitly provided a definition of the IS concept. The concept has evolved from early notions of programme adoption—such as the “extent and rate of adoption” described in 1992—to more refined, multidimensional constructs that include the quantity, quality, dose, and fidelity of implementation. Overall, with minor variations, most definitions in the reviewed studies, trace their origins to a 2011 working paper [19] and a 2012 literature review [11] on IS. The non-IS-adherent studies did not seem to have a common originating source of their definition, while the IS-adherent cited the 2012 literature review and the 2011 working paper. Some definitions focused specifically on measuring IS from the supplier’s perspective (i.e. programme delivered) rather than recipient’s (i.e. programme received). There was inconsistency regarding whether quality is considered a separate dimension or an inherent part of IS. The seminal 2011 paper [19] emphasised collecting data on processes and outputs, while the 2012 review [11] focused on inputs and processes, with an emphasis on linking dose-response relationship. More recent conceptualisations have expanded IS into a broader input-process-output framework, allowing for a more comprehensive measurement [13].

Overall, approximately half of the definitions (48.4%, *n* = 30) included terms such as intensity, extent, quantity, strength, dose, or degree. Around a third (33.9%, *n* = 21) emphasised processes or activities, using terms such as training or efforts. One in four definitions reflected dimension of outputs/results, including quality or service use, while similar proportion (21.0%, *n* = 13) mentioned inputs like resources, supplies, tools, equipment, structure, or resources. Notably, only a few (21.0%, *n* = 13) definitions were goal-oriented, explicitly describing how implementation efforts contribute to intended outcomes.

Our review identified a notable differences between how IS is defined and how it’s measured – as certain dimensions were not explicitly stated in the definitions but they were reflected in actual measurement: strength/quantity (96.8%, *n* = 60), inputs/resources (82.3%, *n* = 51), activities/processes (88.7%, *n* = 55), outputs/results (51.6%, *n* = 32), and goal-oriented (37.1%, *n* = 23).

Broadly, the IS concept serves two main purposes: (a) programme-focused improvements, which involved quantifying implementation efforts (74.2%, *n* = 46) – either as an overall measure or across different programme components [40] - examining IS across geographies [15, 41, 42] and over time [17, 43], and identifying factors influencing IS [44, 45]; and b) outcome-focused improvements, which aimed at exploring the relationship between IS and programme outcomes [10, 17, 45, 46]. The latter was less commonly found in the reviewed studies (56.5%, *n* = 35). Regarding conceptual frameworks, 41.9% (*n* = 26) of studies used a logic model to define and measure IS, followed by 11.3% (*n* = 7) that applied the WHO M&E Framework for health system and used national/international guidelines each. A smaller number drew on the Quality of Care Framework or used multiple framework to inform IS measurement (Table 5).

The comparative analysis demonstrated substantive and statistically significant differences in the definitional dimensions covered between IS-adherent and non-IS-adherent studies. As compared with non-IS-adherent studies, IS-adherent studies more frequently incorporated dimensions related to strength/quantity (76.9% vs. 27.8%), inputs/resources (34.6% vs. 11.1%), processes/activities (50.0% vs. 22.2%), outputs/results (38.5% vs. 13.9%), and goal orientation (34.6% vs. 11.1%). In contrast, no statistically significant differences were observed between the two groups regarding the dimensions captured within the measurement approaches themselves. The only exception was goal-oriented measurement, which was more commonly reported in IS-adherent studies (57.7%) compared with non-IS-adherent studies (27.8%) (*p* = 0.018). Furthermore, IS-adherent studies placed greater emphasis on improving health outcomes (61.5% vs. 52.8%), whereas a relatively higher proportion of non-IS-adherent studies focused on quantifying implementation efforts (80.6% vs. 65.4%). However, these differences were not statistically significant (*p* > 0.05). No significant differences were identified between the two groups in the use of guiding frameworks for IS measurement. The logical model was the most commonly used framework in both groups. The WHO monitoring and evaluation (M&E) framework for health systems emerged as the second most frequently used framework among IS-adherent studies, whereas national and international guidelines were the second most common approach among non-IS-adherent studies.

**Table 5:** Conceptualisation of implementation strength (*N* = 62)

Conceptualisation aspects	Non-IS-adherent studies (*N* = 36)		IS-adherent studies (*N* = 26)		*P*-value for percentage difference	Overall Results (*N* = 62)	
	*n*	%	*n*	%		*n*	%
**Dimensions emerged from definitions**							
Strength/Quantity	10	27.8	20	76.9	< 0.001	30	48.4
Inputs/Resources	4	11.1	9	34.6	0.025	13	21.0
Processes/Activities	8	22.2	13	50.0	0.023	21	33.9
Outputs/Results	5	13.9	10	38.5	0.026	15	24.2
Goal-oriented	4	11.1	9	34.6	0.025	13	21.0
**Dimensions emerged from measurement**							
Strength/Quantity	34	94.4	26	100.0	0.222	60	96.8
Inputs/Resources	28	77.8	23	88.5	0.227	51	82.3
Processes/Activities	31	86.1	24	92.3	0.447	55	88.7
Outputs/Results	19	52.8	13	50.0	0.829	32	51.6
Goal-oriented	10	27.8	15	57.7	0.018	25	40.3
**Purpose of Implementation Strength**							
Improve Health Outcomes	19	52.8	16	61.5	0.492	35	56.5
Quantify Implementation Efforts	29	80.6	17	65.4	0.178	46	74.2
**Guiding framework or reference for IS measurement**							
Logic model	13	36.1	13	50.0	0.274	26	41.9
WHO M&E framework for health system	2	5.6	5	19.2	0.093	7	11.3
National/International guidelines	7	19.4	0	0.0	0.017	7	11.3
Quality of Care Framework	3	8.3	3	11.5	0.674	6	9.7
Others (self-developed or multiple references)	3	8.3	2	7.7	0.927	5	8.1
Not reported	8	22.2	3	11.5	0.277	11	17.7

### Pre-requisites

Our concept analysis identified several prerequisites for the application of IS, broadly categorised into necessary pre-requisites, which are essential for applying the concept effectively, and preferred pre-requisites, which enhance the comprehensiveness and rigor of its application. The necessary pre-requisites were reported in all studies that included a programme or an intervention and anticipated variability in programme implementation. In terms of the preferred pre-requisites, a programme framework (e.g. logic model or standard guidelines) to guide conceptualisation and measurement, availability of routine IPO-related data in routine health information system were emphasised. A few of studies also highlighted the need to adjust for potential confounders when assessing dose-response relationships.

### Measurement

**Fig. 2:**
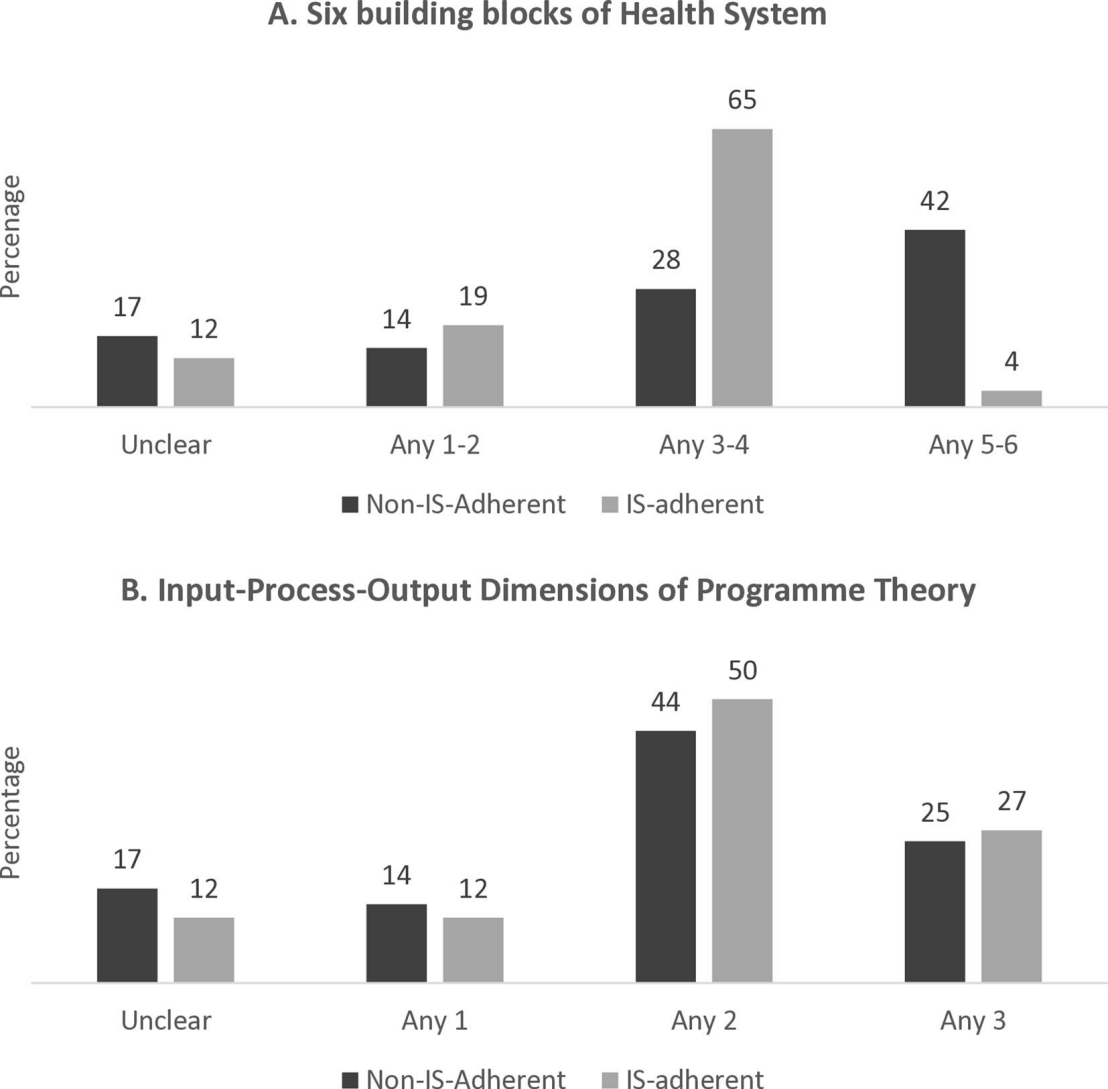
Coverage of IS indicators according WHO Monitoring & Evaluation Framework for Health System (*N* = 62)

Figure 2 compares the coverage of health system components in IS measurement across IS-adherent and non-IS-adherent studies. The non-IS-adherent studies were more comprehensive, 42% covering five to six components, while 65% of IS-adherent studies covered only three to four components. Coverage of the IPO framework was similar, with about half of both groups covering two dimensions.

Table 6 highlights the most commonly used health system and IPO components in measuring IS. Overall, leadership/governance and service delivery were the most frequently included, each in 72.6% (*n* = 45) of studies, while health financing was the least represented, appearing in just 21.0% (*n* = 13). On average, studies used 19 indicators (SD = 21.6), with the highest from service delivery (mean = 6.2 ± 7.0) and the lowest from health financing (mean = 0.4 ± 1.0). Within the IPO framework, process indicators were most common—used in 80.6% of studies with a mean of 12.6 ± 13.8 indicators—followed by inputs (74.2%) with an average of 6.3 ± 8.7 indicators.

The coverage of health system components was consistently lower in IS-adherent studies compared to non-IS-adherent ones. These studies most frequently included workforce (73.1%, *n* = 19) and leadership/governance (69.2%, *n* = 18), while information systems (19.2%, *n* = 5) and health financing (11.5%, *n* = 3) were largely neglected. Even in non-IS-adherent studies, health financing remained the least represented component (27.8%, *n* = 10).

IS-adherent studies also used significantly fewer indicators overall than non-IS-adherent studies (mean = 11.0 vs. 25.1), with the exception of the supplies and equipment component. While the coverage was similar, they showed significantly lower indicator counts across all IPO components: inputs (mean = 3.9 vs. 8.1, *p* = 0.055), processes (mean = 6.2 vs. 17.4, *p* = 0.001), and outputs (mean = 0.7 vs. 1.8, *p* = 0.057).

**Table 6:** Distribution of IS indicators according to WHO Evaluation Framework

Components	Non-IS-adherent studies (*N* = 36)				IS-adherent studies (*N* = 26)				*P*-value for mean difference	Overall Results (*N* = 62)			
	Covered in IS measurement		Mean	SD	Covered in IS measurement		Mean	SD		Covered in IS measurement		Mean	SD
	*n*	%			*n*	%				*n*	%		
WHO Health System Building Blocks*													
Leadership / Governance	27	75.0	6.8	8.4	18	69.2	2.4	2.2	0.009	45	72.6	4.9	6.8
Workforce	24	66.7	3.8	5.6	19	73.1	1.6	1.2	0.04	43	69.4	2.8	4.4
Service delivery	31	86.1	8.4	8.0	15	57.7	3.4	4.0	0.004	46	74.2	6.2	7.0
Information system	26	72.2	5.1	6.1	5	19.2	0.4	1.0	< 0.001	31	50.0	3.0	5.2
Supplies and equipment	23	63.9	2.8	4.5	17	65.4	3.1	4.4	0.791	40	64.5	2.9	4.5
Financing**	10	27.8	0.5	1.2	3	11.5	0.1	0.3	0.046	13	21.0	0.4	1.0
Input-Process-Output Framework*													
Input	26	72.2	8.1	10.9	20	76.9	3.9	3.7	0.055	46	74.2	6.3	8.7
Process	28	77.8	17.4	16.3	22	84.6	6.2	5.2	0.001	50	80.6	12.6	13.8
Output	16	44.4	1.8	2.7	11	42.3	0.7	1.1	0.057	27	43.5	1.3	2.2
Outcome	2	5.6	0.0	0.0	1	3.8	0.0	0.0	NC	3	4.8	0.0	0.0
**Total Indicators**	**-**	**-**	**25.1**	**26.0**	**-**	**-**	**11.0**	**7.5**	**0.006**	**-**	**-**	**19.3**	**21.6**

Note: 5 studies did not report exact indicators and 4 studies did not report any details even about measurement components. These studies were excluded from the denominate for the calculation of percentage as appropriate

NC: Not calculated

An overwhelming majority (93.2%, *n* = 55) of the studies across both the groups relied on special surveys to measure IS. With respect to type of indicators, non-IS-adherent studies most frequently employed ordinal indicators (27.6%, *n* = 16), followed by binary (19.0%, *n* = 11) and finite-range continuous indicators (19.0%, *n* = 11). In contrast, IS-adherent studies predominantly used continuous-scale indicators, particularly those with an infinite range (32.7%, *n* = 17), followed by probability-based indicators (28.8%, *n* = 15) and binary indicators (21.2%, *n* = 11). Although probability-based indicators made up the largest proportion of indicators within individual studies, discrete or continuous indicators with an infinite range were used minimally—typically only 1 or 2 within a study (results not shown).

Composite IS indices were less commonly created among IS-adherent studies (50%, *n* = 13) compared to non-IS-adherent ones (91.4%, *n* = 32). In most non-IS-adherent studies, the scale of composite indices were ordinal (44.1%, *n* = 15) and probability-based ranging from 0 to 1 (32.4%, *n* = 11), whereas IS-adherent studies used a mix of ordinal (33.3%, *n* = 6), finite-range (27.8%, *n* = 5) and infinite-range (33.3%, *n* = 6) continuous scales (Table 7).

**Table 7:** Data source and scale of IS indicators

Measurement characteristics	Non-IS-adherent studies (*N* = 36)		IS-adherent Studies (*N* = 26)		Overall Results (*N* = 62)	
	*n*	%	*n*	%	*n*	%
Data source*						
Survey	26	72.2	14	60.9	40	67.8
HMIS	2	5.6	2	8.7	4	6.8
Survey & HMIS	8	22.2	7	30.4	15	25.4
**Characteristic of indicators**						
Scale of IS indicators**						
Discrete/Continuous with infinite range	8	13.8	17	32.7	25	22.7
Probability (0–1)	8	13.8	15	28.8	23	20.9
Binary	11	19.0	11	21.2	22	20.0
Discrete / Continuous with finite range	11	19.0	8	15.4	19	17.3
Ordinal	16	27.6	1	1.9	17	15.5
Not reported	4	6.9	0	0.0	4	3.6
**Created a composite score**						
Yes	32	91.4	13	50.0	45	73.8
No	4	8.6	13	50.0	17	26.2
**If yes**, scale of composite score**						
Ordinal	15	44.1	6	33.3	21	40.4
Probability (0–1)	11	32.4	1	5.6	12	23.1
Discrete/continuous with finite range	6	17.6	5	27.8	11	21.2
Discrete/continuous with infinite range	2	5.9	6	33.3	8	15.4
Binary	0	0.0	0	0.0	0	0.0

* 3 literature review from IS adherent studies were excluded from the denominator as they didn’t report on data source

** Denominator is number of responses, not number of studies

Studies used a variety of statistical techniques to construct a composite indices, with the unweighted percentage (17.3%, *n* = 9) and unweighted sum (15.4%, *n* = 8) being the most commonly used techniques, overall. Among IS-adherent studies, multivariate statistical techniques such as PCA / EFA were most commonly used (25.0%, *n* = 5) followed by unweighted sum (20.0%, *n* = 4) and use of 1–2 key variable(s) (20.0%, *n* = 4) among the pool of indicators. Conversely, unweighted percentage (21.9%, *n* = 7) and weighted percentage (18.8%, *n* = 6) were more commonly employed techniques among non-IS-adherent studies.

Furthermore, half of the studies (51.1%, *n* = 23) did not categorise the composite IS index or in order words used it as a continuous measure, whereas, the remaining studies applied either self-defined criteria (35.6%, *n* = 16) or a percentile-based technique (13.3%, *n* = 6) for categorisation. A similar pattern was observed across both the groups. Only 28.9% (*n* = 13) of the studies reported psychometric properties for the IS index, primarily assessing internal consistency through Cronbach’s alpha, with reported values ranging from 0.62 to 0.96—indicating acceptable to excellent reliability. The reporting of psychometric properties was more prevalent in IS-adherent studies (38.5%, *n* = 5) compared to non-adherent studies (25.0%, *n* = 8) (Table 8).

**Table 8:** Use of statistical techniques for creating a composite IS index and psychometric properties

Measurement characteristics	Non-IS-adherent studies (*N* = 36)		IS-adherent Studies (*N* = 26)		Overall Results (*N* = 62)	
	*n*	%	*n*	%	*n*	%
**Created a composite IS index**						
Yes	32	91.4	13	50.0	45	73.8
No	4	8.6	13	50.0	17	26.2
**Step I: If yes**, statistical techniques used for construction*						
Unweighted percentage	7	21.9	2	10.0	9	17.3
Unweighted sum	4	12.5	4	20.0	8	15.4
Key variable(s) 1/2	3	9.4	4	20.0	7	13.5
Unweighted average	5	15.6	1	5.0	6	11.5
Weighted percentage	6	18.8	0	0.0	6	11.5
PCA / EFA	0	0.0	5	25.0	5	9.6
Weighted Sum	4	12.5	1	5.0	5	9.6
Weighted average	2	6.3	2	10.0	4	7.7
Calculation technique not reported	1	3.1	0	0.0	1	1.9
Multiplication	0	0.0	1	5.0	1	1.9
**Step II: If yes**,** categorisation technique used for composite index**						
Didn’t categorise/used as a continuous index	16	53.1	6	46.2	23	51.1
Self-defined criteria	12	37.5	4	30.8	16	35.6
Percentile-based	3	9.4	3	23.1	6	13.3
**Psychometric Properties**						
Not reported	24	75.0	8	61.5	32	71.1
Reported	8	25.0	5	38.5	12	28.9

* Denominator is number of responses, not number of studies

Note: PCA: Principal Component Analysis; EFA: Exploratory Factor Analysis

### Use of IS measure

Overall, most studies used the ‘health facility’ (33.9%, n = 21) as the primary unit of analysis, followed by ‘healthcare professional’ (24.2%, *n* = 15). When disaggregated by study type, non-IS-adherent studies predominantly used the health facility level (52.8%, *n* = 19), while IS-adherent studies mainly used healthcare professionals (46.2%, *n* = 12) as the unit of analysis. With regard to the type of analysis, IS-adherent studies more frequently applied descriptive analysis of IS measures (50%, *n* = 13), while cross-classification analysis of IS measure was more common among non-IS-adherent studies (80.5%, *n* = 29) (Table 9).

**Table 9:** Statistical techniques used to analyse IS

Analytical characteristics	Non-IS-adherent studies (*N* = 36)		IS-adherent Studies (*N* = 26)		Overall Results (*N* = 62)	
	*n*	%	*n*	%	*n*	%
Unit of analysis*						
Health facility	19	52.8	2	7.7	21	33.9
Healthcare professional	3.0	8.3	12	46.2	15	24.2
Cluster	3.0	8.3	5	19.2	8	12.9
District	1.0	2.8	5	19.2	6	9.7
State	4	11.1	0	0.0	4	6.5
Health facilities	2	5.6	0	0.0	2.0	3.2
Country	1	2.8	0	0.0	1	1.6
Individual, District, Country	0	0.0	1	3.8	1	1.6
NR	0	0.0	1	3.8	1	1.6
Patient	1.0	2.8	0	0.0	1	1.6
Pharmacies	1	2.8	0	0.0	1	1.6
**Performed descriptive analysis of IS index**						
Yes	10	27.8	13	50.0	23	37.1
No	26	72.2	13	50.0	39	62.9
**If yes**,** how**						
Individual-indicator	4	40.0	12	92.3	16	69.6
Not reported	1	10.0	1	7.7	2	8.7
Composite	1	10.0	0	0.0	1	4.3
Individual & composite	1	10.0	0	0.0	1	4.3
Sub-domain & Composite	1	10.0	0	0.0	1	4.3
Individual, Sub-domain & Composite	1	10.0	0	0.0	1	4.3
Sub-domain	1	10.0	0	0.0	1	4.3
**Performed cross-classification analysis of IS index**						
Yes	29	80.5	17	65.4	46	74.2
No	7	19.5	9	34.6	16	25.8
**If yes**,** how**						
Composite	11	37.9	5	29.4	16	34.8
Individual	6	20.7	10.0	58.8	16	34.8
Sub-domain	6	20.7	1	5.9	7	15.2
Individual & sub-domain	3	10.3	0	0.0	3	6.5
Not reported	1	3.4	1.0	5.9	2	4.3
Composite & sub-domain	2	6.9	0	0.0	2.0	4.3

* Multiple response possible

### Consequences: association between IS and outcomes

Only half of IS-adherent studies (50%, *n* = 13) and a quarter of non-IS-adherent studies (25.7%, *n* = 9) assessed the association between IS indices and health outcomes. Among these, 90% (*n* = 20) reported a positive association, with consistent findings across both groups. Most studies were rated as ‘high’ quality, while about one-third were of ‘acceptable’ quality. Notably, the two studies that found no significant association [47, 48] were both rated ‘acceptable’ and used limited IS measures, relying on only two to four overlapping indicators (Table 10).

**Table 10:** Association between IS and health outcomes

Analytical characteristics	Non-IS-adherent studies (*N* = 36)		IS-adherent Studies (*N* = 26)		Overall Results (*N* = 62)	
	*n*	%	*n*	%	*n*	%
**Performed associational analysis with outcome**						
Yes	9	25.0	13	50.0	22	35.5
No	27	75.0	13	50.0	40	64.5
**If yes**,** significance of association (statistical)**						
No association	1	11.1	1	7.7	2	9.1
Positive	8	88.9	12	92.3	20	90.9
Negative	0	0.0	0	0.0	0	0.0

### Research quality assessment

We assessed the quality of 22 studies exploring the dose-response relationship between IS and health outcomes, based on design, bias, comparability, and methodological rigour. The majority were cross-sectional (70%, *n* = 14), with four cohort and two experimental studies. Just over half (54.5%, *n* = 12) were rated as high quality, while 31.8% (*n* = 7) were acceptable and 13.6% (*n* = 3) were below acceptable standards. Common limitations included inadequate measurement of IS—such as incomplete coverage of programme components and lack of psychometric validation—and insufficient adjustment for confounders in statistical analyses.

## Discussion

IS, a cardinal concept in the field of implementation science, is used to assess how implementation of health services and programmes influences health outcomes. It offers a unique opportunity to determine how much implementation efforts are required to achieve a meaningful level of change in outcomes [10]. As the use of IS became widespread, the need for conceptual clarification and systematic application became increasingly evident. We explored how IS is conceptualised and measured, and examined whether stronger IS is associated with better outcomes.

Despite the evolution of the IS concept over time, IS remains vaguely and inconsistently conceptualised, often with a marked discrepancy between how it is defined and measured within studies. The concept has primarily been applied to programmes implemented in real-life settings; however, no study has applied it to routine health services aimed at strengthening the healthcare system. Studies conceptually-grounded in 2011 and 2012 IS guidance differed notably from their counterparts: they used fewer but more relevant indicators, applied advanced statistical techniques to construct composite indices, and assessed dose-response relationships. Yet, overall, the measurement of IS, often fell short of capturing the full scope of programmes as per the WHO evaluation framework (the six building blocks and IPO framework). Measurement practices also varied widely, often conflating IS with related concepts like fidelity. Most studies did not examine the dose-response (IS-outcome) relationship, but those that did consistently found a strong positive association. While half of the studies were high quality, most lacked valid and reliable composite IS index.

**Terminology of IS**: Several terminologies are used to label this concept, including: strength, intensity, rate, extent, degree, and. For example, “rate” refers to a quantity or amount considered in relation to or measured against another quantity or amount [49]. In epidemiology, this measure requires a time factor for its computation [50, 51]. The terms “implementation extent” and “degree” have similar meanings in this context, assessing the amount, point, or limit to which something extends toward fullness [52, 53], making them more suitable for fidelity. The term “level” refers to the stage or hierarchy of activities, which inherently implies that stage 2 is better than stage 1 based on differing characteristics. However, differentiation in characterizations within implementation intensity is unnecessary. “Implementation strength” and “Implementation intensity” were the two most commonly and interchangeably used terms found in the literature to label the IS concept. The term “strength” primarily connotes the robustness of programme implementation, which not only carries an element of subjectivity but also holds different meanings across disciplines – for instance, referring to the power to resist or exert force [54]. Scott and colleague also referred ‘strength’ (specifically ‘strength of intervention’) as a combined construct of several dimension that include purity, specificity, dosage, frequency, and quantity [55]. The term “implementation intensity”, by contrast, refers to the frequency, magnitude, or quantity of efforts, activities, or use of resources [55]. Since, the primary purpose of IS is to capture quantifiable dimension (Table 7), “implementation intensity” may offer greater conceptual precision for this construct. Nonetheless, we recognise it warrants scholarly deliberation, and we invite experts in the field to consider whether the term better captures the construct as it is currently operationalised in practice. The use of consistent terminology will: enhance search accuracy, improve indexing & discoverability, bring consistency in usage (clarity & harmonisation) [56]. Similarly, adding a clear definition in the publications leads to better understanding, clarity, and comparison across studies [56].

**Conceptualisation and purpose of IS**: The IS conceptualisation evolved to a more multidimensional construct over time, with most definitions stemmed from a working paper [19] and a literature review on the concept [11]. Our review identified key conceptual and measurement ambiguities—for example, whether IS should be measured from the supplier side (quantity delivered) or recipient side (quantity received) [13, 19]; whether quality is distinct from or integral to IS; and whether measurement indicators should reflect input–process or process–output. Implementation effort can be assessed through either quantity delivered or quantity received; however, we argue that the former is easier and less resource-intensive, as it falls within the scope of health system monitoring and relies on routine HMIS data. We also consider quality a critical output-level indicator, strongly linked to health outcomes, and therefore believe it should be an integral part of IS. Regarding indicator types within the Input-Process-Output (IPO) framework, we contend that IS measurement should encompass all levels to ensure a comprehensive understanding of implementation efforts [13]. Despite its vague definitional characterisation, there is a general consensus on two broad purposes of the IS concept: quantification of implementation efforts (overall and across different programme domains), and examining its association with desired outcomes. However, the latter is often overlooked in both definitions and application. In our view, IS primary value lies in showing how implementation efforts influence outcomes, guiding programme adjustments to optimise impact [10]. Drawing on the dimensions most consistenly reflected across both definitions and measurement practices identified in this review, we propose the following working definitions: *“Implementation Strength of routine healthcare services or a programme refers to an epidemiological and statistical technique that quantifies implementation efforts – ranging from administrative inputs to service providers*,* system process support*,* and output – as a summary measure*,* aimed at understanding implementation variability and establishing a dose-response relationship with healthcare system or programme targets”.* This definition is intentionally grounded in the input-process-output framework, retains the quantitative character, and sufficiently broad to accommodate diverse health system contexts.

### Components of IS

A review of measurement against the WHO evaluation framework revealed that in most studies, the IS measurement was primarily based on a limited set of process indicators (at times only 1 or 2) that focused on certain components of health system. From a programmatic perspective, such narrowly focused measurements limits the ability to comprehensively understand how all programme components interact to achieve intended results, thereby hindering adequate improvements efforts [57]. Furthermore, from an epidemiological standpoint, such compromised measurement may also introduce residual confounding, particularly when linking implementation efforts to health outcomes [50, 51]. To build a comprehensive understanding of programme implementation, measurement should encompass all components of health system. We suggest the use of WHO evaluation framework – encompassing all six building blocks and the IPO dimension - can help standardise IS conceptualisation and measurement methodologies which will enable more meaningful comparisons [31].

### Measurement of IS

**Data source –** Our review noted that an overwhelming majority of the studies relied heavily on special surveys for IS measurement. This may be due to the unavailability or poor quality of the requisite data [58, 59]. Since IS is primarily a monitoring approach meant to guide implementation, strengthening HMIS in low-resource settings is essential to ensure availability of data for IS measurement. This would have several benefits: (a) reduce reliance on resource-intensive surveys [60]; (b) use of HMIS data for decision making will galvanise continuous efforts to gradually improve data quality [61], and (c) availability of HMIS data will increase the feasibility of embedding this monitoring approach within health system, thereby allowing health system stakeholders to assess temporal trends in IS and associating it with outcomes. In contrast, special survey should be the least preferred choice and only be considered in scenarios where requisite HMIS data are either not available or of poor quality [58, 59].

**Indicator Scale -** Despite a unique theoretical advantages of measuring IS – that is to meticulously quantify implementation efforts, most studies used binary or ordinal (including perception-based) indicators rather than factual continuous indicators, resulting in a compromised IS index. The use of categorical variables introduces several statistical limitations, including loss of granular details, reduced statistical power, increased risk of misclassification [51], and lower precision. While binary and categorical measurement do indicate some level of strength, this limits the ability to derive meticulous insights into implementation efforts which can lead to oversimplified decision-making, and hinder comparability across studies due to inconsistent categorisation thresholds [62]. Furthermore, use of binary indicators of service availability and readiness and analysing each indicator individually against benchmarks at the health worker or facility level [63] may risk diluting its unique identity and conflating the concept with implementation fidelity. To maintain a clear distinction and optimum use of the concept, it is important to use continuous indicators (where possible) that in essence reflect the amount or quantity of efforts. Lastly, we prefer the use of factual /objective indicators for IS measurement over perception-based indicators as they reflect the true quantity of efforts, thereby allowing decision makers to precisely determine what needs to change and to what extent in relation to outcomes. Importantly, objective measures can be verified, which makes them reliability and thus help ensure transparency and accountability [62].

**Composite index -** Most studies used unweighted average and/or sum to create composite indices without reporting their psychometric properties. This raises concerns about reliability and validity of composite indices. Moreover, unweighted composite indexing may imply all variables carry equal important; however, when variables are grouped into a dimension and then aggregated, it can result in unequal weighting – giving more weights to dimensions with more variables – and lead to an imbalanced IS index structure [64]. The use of more advanced statistical techniques like principal component analysis and factor analysis was more common in IS-adherent studies. While such techniques are considered data-driven, they are complex and are difficult to reproduce in routine use by programme teams as it requires specialised skills [65]. An unreliable or invalid IS index can lead to measurement bias, reduced reproducibility, inconsistent comparisons across studies, misclassification of IS levels, and inaccurate or misleading dose-response relationships [66]. While creating a composite index is a complex process with no universally valid method for its application, its validity depends on the strategic objective of the research [64, 65]. To improve measurement validity, researchers should adopt more robust statistical techniques, such as Principal Component Analysis (PCA) or Factor Analysis, to develop standardised and validated indices for IS assessment. This should be complemented by a simplified approach for programme implementers for routine use [64, 67].

**Unit of analysis -** Health facility and healthcare professionals emerged as the most common unit of analysis. While analysing IS at the health facility level is valid, the choice of unit of analysis should align with the intended user. Given that data can be used at any levels, we stressed on analysing IS at two key levels. First, IS data should be measured or aggregated at the district level and linked to clearly defined health outcomes. This approach is especially valuable for higher autonomous administrative units (e.g. provincial, divisional), and even allow district health teams to assess their implementation efforts and outcomes in comparison with other districts. District-level will likely enable the use of population-level outcome data from sources like MICS and other household surveys. Second, health facilities can be used as the unit of analysis to examine the association between IS and facility-level outputs (e.g., key performance indicators such as services provided). Although identifying direct population-level outcomes at this level may be challenging without survey data, this approach is highly relevant for district administrators aiming to enhance facility performance. In such cases, facility outputs should be selected based on their strong link to population-level outcomes, allowing robust inferences about potential change in the population based on facility-level outputs. For example, inferences about immunization coverage and contraceptive use could be reliably made based on services data on child vaccination and contraceptive services, respectively. In a nutshell, we emphasise that IS should ideally be analysed at an autonomous administrative units where decision making and resource allocation occurs. This shift would enable more meaningful planning and decision-making [68].

#### Pre-requisites

A fundamental pre-requisite for the application of IS is a programme being implemented in a real-life setting. The application of IS is not suited for research studies where fidelity is a more appropriate concept. However, a notable observation was that none of the IS application was done in routine health services within health system, apart from health programme which are generally time-bound. In our view, the concept is equally applicable for monitoring routine health services within health systems and future studies should demonstrate the use of IS on routine health services aiming to strengthen healthcare systems striving to achieve regional and national commitments.

**Consequences -** A core purpose of measuring IS is to link implementation efforts with programme outcomes to drive improvements. Yet, most studies did not assess this relationship, often relying on special cross-sectional designs, descriptively analysing each indicators (or domains) at health worker or facility level without composite scores and linking with relevant outcomes. In our view, this approach is not fully aligned with the unique theoretical foundation of the IS concept and does not leverage its full potential. Since IS is a relative measure, it should be analysed in relation to other variables—such as the factors influencing IS or relevant outcomes—to maintain its distinct value. Only a few studies—even among those following conceptual guidance on IS—adhered to this approach [17, 46, 69]. Importantly, nearly all studies reported a significant positive association between IS and health outcomes, underscoring the concept’s value and its potential to drive improvements through objective assessment guiding strategic operational planning.

### Strengths and limitations

We believe that combining the rigor of a systematic review with the depth of concept analysis, and proposing an integrated framework, is a key contribution to furthering the debate on this concept. However, this research has some limitations. First, the classification of indicators according to the IPO framework follows our operational definition, but depending on the scope of the programme and contextual factors, indicators may need to be reclassified (e.g., from process to output). Second, while the Walker and Avant approach is widely used, it is sometimes viewed as positivistic, rigid, and reductionist [70], adopting an entity view of the concept rather than a relational one [70, 71]. Third, this review focused solely on public health programmes integrated with the healthcare system, excluding interventions in education and social services. Finally, our review only included English-language articles due to our familiarity with the language, which may introduce potential publication bias, particularly affecting generalizability and contextual insights. However, evidence on whether such exclusions significantly alter estimates or conclusions is mixed [72, 73].

## Conclusion

Our review offers valuable insights into the conceptualisation, measurement and practical use of the IS concept in health system settings. The review comprehensively mapped existing confusion, critically examined its strengths and limitation, and offered a scientifically grounded resolution aimed at standardising the conceptualisation and application of IS, thereby enabling consistent comparisons across settings. The concept has predominantly been applied to health programmes whereas no study applied in the context of routine health services. Furthermore, our findings provide strong evidence of a significant positive association between IS and health system outcomes, reinforcing the value and potential of this approach in strengthening health services and systems through objective measurement and strategic implementation planning. We believe this review will stimulate further discussions among health system researchers, international funding agencies, and policymakers, potentially leading to an international consensus and roadmap for embedding this concept within health systems globally as a routine monitoring and planning approach for achieving programme outcomes and guiding scaling up efforts.

Our review offers key recommendations to advance IS research and promote its use within health systems. Building on the broader guidance provided, there is a need to develop standardised, validated IS indices for various health themes, with an emphasis on viable, cost-effective methods that can be integrated into routine health systems and used by administrators for decision-making. Future applied research should explore how IS can be implemented and used in real-world settings across diverse health themes and administrative levels. From health system perspectives, strengthening health management information systems (HMIS) in low-resource settings is essential to ensure availability of quality data for IS measurement. Health administrators should consider embedding IS as a routine monitoring approach to oversee programme implementation. At the policy level, IS findings inform the development of national health strategies and implementation plans, including the scale-up of evidence-based interventions.

## Supplementary Information

Below is the link to the electronic supplementary material.

Supplementary Material 1

## Data Availability

This systematic review is based on published research articles that are publicly available. We have, however, add the detailed list of articles that appeared through a database search.
