# Supplementary material for: Conceptualisation and application of implementation strength in public health programmes within health systems: a systematic review
Source: BMC Health Serv Res. 2026 Jun 9;26:1129. doi: 10.1186/s12913-026-14856-w (PMC13479942; doi:10.1186/s12913-026-14856-w)
Supplement: Supplementary file 1 [file 12913_2026_14856_MOESM1_ESM.docx]

**Search strategy:**

| **S.no** | **Search strategy [date search executed]** | **Results** |
| --- | --- | --- |
| **1** | **PUBMED [10^th^ September 2022]** |  |
| **1.5** | ("implementation strength"[TIAB] OR "implementation intensity"[TIAB] OR "implementation extent"[TIAB] OR "implementation degree"[TIAB] OR "implementation level"[TIAB] OR "implementation rate"[TIAB] OR "measure implementation"[TIAB] OR "measuring implementation"[TIAB] OR "measured implementation"[TIAB] OR "measures implementation"[TIAB] OR "evaluate implementation"[TIAB] OR "evaluates implementation"[TIAB] OR "evaluated implementation"[TIAB] OR "evaluating implementation"[TIAB] OR "assess implementation"[TIAB] OR "assesses implementation"[TIAB] OR "assessed implementation"[TIAB] OR "assessing implementation"[TIAB] OR "examine implementation"[TIAB] OR "examines implementation"[TIAB] OR "examined implementation"[TIAB] OR "examining implementation"[TIAB]) AND ((humans[Filter]) AND (english[Filter]) AND (1970:2022[pdat])) | **937** |
| **2** | **SCOPUS [10^th^ September 2022]** |  |
| **2.6** | TITLE-ABS-KEY ( ( "implementation strength" OR "implementation intensity" OR "implementation extent" OR "implementation degree" OR "implementation level" OR "implementation rate" OR "measure implementation" OR "measures implementation" OR "measuring implementation" OR "measured implementation" OR "evaluate implementation" OR "evaluates implementation" OR "evaluating implementation" OR "evaluated implementation" OR "assess implementation" OR "assesses implementation" OR "assessing implementation" OR "assessed implementation" OR "examine implementation" OR "examines implementation" OR "examining implementation" OR "examined implementation") ) AND ( PUBYEAR > 1969 ) AND ( LIMIT-TO ( SUBJAREA , "MEDI" ) ) AND ( LIMIT-TO ( LANGUAGE , "English" ) ) | **1,857** |
| **3** | **ERIC [September 21, 2022]** |  |
| **3.3** | "implementation strength" OR "implementation intensity" OR "implementation extent" OR "implementation degree" OR "implementation level" OR "implementation rate" OR "measure implementation" OR "measures implementation" OR "measuring implementation" OR "measured implementation" OR "evaluate implementation" OR "evaluates implementation" OR "evaluating implementation" OR "evaluated implementation" OR "assess implementation" OR "assesses implementation" OR "assessing implementation" OR "assessed implementation" OR "examine implementation" OR "examines implementation" OR "examining implementation" OR "examined implementation"  Limiters - Published Date: 19700101-20221231; Hidden NetLibrary Holdings  Narrow by Language: - english  Search modes - Boolean/Phrase | **1603**  **[1418 after auto-removal of duplicates by ERIC]** |
| **4** | **GOOGLE SCHOLAR [September 21, 2022]** |  |
| **4.1** | "implementation strength" OR "implementation intensity" OR "implementation level" OR "implementation rate" OR "measure implementation" OR "evaluate implementation" OR "evaluate implementation" OR "assess implementation" OR "examine implementation"  Time: 1970 – 2022  Sort by relevance  English | **17200**  **Taken first 200** |
| **4.2** | "implementation strength" OR "implementation intensity" OR "implementation extent" OR "implementation degree" OR "implementation level" OR "implementation rate" | **16,900** |
| **5** | **Bill & Malinda Gates Foundation [09^th^ December 2024]** |  |
| **6.1** | "implementation strength" OR "implementation intensity" OR "implementation extent" OR "implementation degree" OR "implementation level" OR "implementation rate" | **0** |
| **6** | **Doctors without Borders (Medecins sans Frontiers) [09^th^ December 2024]** |  |
| **6.1** | "implementation strength" | **0** |
| **7** | **The Kaiser Family Foundation** [**http://www.kff.org/**](http://www.kff.org/) **[09^th^ December 2024]** |  |
| **7.1** | "implementation strength" | **19** |
| **8** | **Open Society Foundations** [**http://www.soros.org/**](http://www.soros.org/) **[09^th^ December 2024]** |  |
| **8.1** | "implementation strength" | **0** |
| **9** | **Partners in Health** [**http://www.pih.org/publications/**](http://www.pih.org/publications/) **[09^th^ December 2024]** |  |
| **9.1** | "implementation strength" | **0** |
| **10** | **Popline** [**http://www.popline.org/**](http://www.popline.org/) **[09^th^ December 2024]** |  |
| **10.1** | "implementation strength"  "implementation strength" OR "implementation intensity" OR "implementation extent" OR "implementation degree" OR "implementation level" OR "implementation rate" | **20** |
| **11** | **Public Health Institute** [**http://www.phi.org/resource_library/index.html**](http://www.phi.org/resource_library/index.html) **[09^th^ December 2024]** |  |
| **11.1** | "implementation strength" | **96** |
| **12** | **Social Policy and Practice** [**http://scholar.google.co.uk/**](http://scholar.google.co.uk/) **[09^th^ December 2024]** |  |
| **12.1** | "implementation strength" |  |
| **13** | **United Nations [09^th^ December 2024]** |  |
| **13.1** | http://www.un.org/en/  "implementation strength" OR "implementation intensity" OR "implementation extent" OR "implementation degree" OR "implementation level" OR "implementation rate" | **0** |
| **13.2** | <http://www.unfpa.org/public/>  "implementation strength” | **0** |
| **13.3** | <http://www.unaids.org/en/>  "implementation strength" OR "implementation intensity" | **6** |
| **13.4** | <http://www.undp.org/content/undp/en/home.html>  "implementation strength" OR "implementation intensity" OR "implementation extent" OR "implementation degree" | **64** |
| **13.5** | <http://www.unodc.org/>  "implementation strength" OR "implementation intensity" OR "implementation extent" OR "implementation degree" OR "implementation level" OR "implementation rate" | **0** |
| **14** | **World Health Organization** [**http://www.who.int/en/**](http://www.who.int/en/) **[09^th^ December 2024]** |  |
| **14.1** | "implementation strength" | **0** |
